# *Clostridiaceae* in Cancer Management

**DOI:** 10.32604/or.2026.074037

**Published:** 2026-06-16

**Authors:** Julia Banaszkiewicz, Paweł Krawczyk, Tomasz Grenda, Anna Grenda

**Affiliations:** 1 Student Scientific Circle at the Chair of Pneumonology, Oncology and Allergology, Medical University of Lublin, Lublin, Poland; 2 Immunology and Genetics Laboratory, Medical University of Lublin, Lublin, Poland; 3 Department of Food and Feed Microbiology, National Veterinary Research Institute, Puławy, Poland

**Keywords:** Cancer, treatment, *Clostridiaceae*, spores

## Abstract

Unfavorable epidemiological forecasts indicating a significant increase in cancer incidence and mortality, as well as limitations of traditional cancer treatment methods, prompt the search for new, more effective therapeutic strategies. In response to the difficulties in treating cancer resulting from the significant heterogeneity of the tumor microenvironment and the presence of hypoxic and necrotic zones, anaerobic bacteria from the *Clostridiaceae* family, particularly those of the *Clostridium* genus, are attracting increasing interest. These bacteria can selectively grow in hypoxic areas of tumors while showing no affinity for healthy tissues. An additional advantage of these bacteria is their ability to produce toxins and enzymes that enable the lysis of cancer cells and activate the immune response. One of the most promising and distinctive strains is *Clostridium novyi*-NT, which lacks virulence factors and, in studies, has been shown to induce a strong cytolytic response. Modern genetic engineering has enabled the modification of *Clostridiaceae* bacteria to express enzymes that activate prodrugs, thereby significantly reducing systemic toxicity while targeting tumor tissue. Combining *Clostridiaceae* spore therapy with conventional treatments, such as chemotherapy, radiotherapy, or immunotherapy, significantly enhances their effectiveness, resulting in a highly beneficial synergistic therapeutic effect. The purpose of this review is to discuss the role and potential of *Clostridiaceae* bacteria in innovative anticancer therapies.

## 1 Introduction

### Cancer Burden, Limitations of Standard Therapy, Clostridium Anticancer Treatment

Cancer is currently one of the most significant problems and challenges for public health and modern medicine, being the leading cause of death in both more and less developed countries. The World Health Organization (WHO) reports that the burden of cancer incidence and mortality is steadily increasing. It is estimated that by 2050, the number of cancer cases will dramatically increase by as much as 77%, while the number of deaths will increase by almost 90% compared to 2022 [1,2]. It is due to increased exposure to environmental risk factors, including radiation, pollution, and behavioral factors such as smoking, physical inactivity, and an unhealthy diet [3,4,5].

The growing scale of the problem marks the need to search for novel therapeutic interventions that increase the effectiveness of cancer treatment while reducing the incidence of side effects. Difficulties in cancer therapy stem primarily from the biological complexity of tumors. Solid tumors exhibit considerable genetic and metabolic heterogeneity. The result of their specific structure is the ability to rapidly develop resistance to the drugs or evade the immune response [6,7,8]. The formation of a chaotic and abnormal network of blood vessels in the tumor environment leads to the development of extensive areas of hypoxia and necrosis at the tumor’s center [9,10,11,12,13]. The resulting zones of hypoxia prevent the effective penetration and action of chemotherapeutics and radiotherapy [10,13,14] and also contribute to the selection of more aggressive cell phenotypes by activating adaptive mechanisms, such as the HIF-1α (Hypoxia-Inducible Factor 1α)-dependent pathway [11,15,16].

Standard therapies such as surgery, radiation therapy, immunotherapy, molecularly targeted therapy, and chemotherapy, while widely used for treatment, face various limitations. These include toxic side effects resulting from damage to healthy tissues [17]. Even the mentioned immunotherapy, as one of the most modern treatment methods, yields outstanding results in extending the progression-free survival (PFS) and overall survival (OS), but is not effective in all patients; the reasons for this are not fully understood [18,19,20]. The ineffectiveness of targeting subpopulations of cancer cells in hard-to-reach regions of the tumor poses a significant challenge, as the difficulty of achieving selective action within tumor tissue becomes a major issue [21,22]. The development of innovative therapies would enable overcoming these barriers and targeting the tumor’s biological features, thereby increasing treatment effectiveness. In recent decades, therapies that exploit the biological selectivity of certain microorganisms, particularly anaerobic bacteria of the *Clostridium* genus, have gained popularity. These are Gram-positive bacilli belonging to the *Firmicutes* type, which are capable of forming extremely resistant spores under unfavorable conditions. In contrast, in oxygen-limited environments such as the central zones of solid tumors, germination into vegetative cells and intensive multiplication occur [23,24]. After intravenous administration, bacterial spores germinate selectively in the tumor environment and remain inactive in healthy tissues, minimizing damage to normal cells [25].

William B. Colley was the first to investigate the possibility of treating solid tumors with a bacterial mixture later called “Coley’s toxin”. These historical observations, dating back to the late 19th century, served as the basis for subsequent research on lytic bacteria, including species from the family *Clostridiaceae*. These aroused particular interest in the mid-20th century. Although the results remained inconclusive and did not achieve complete tumor remission, they provided a crucial impetus for further studies and the continued development of this innovative therapeutic strategy [26].

Currently, research is focused on non-pathogenic strains or those lacking virulence factors. One of these is *Clostridium novyi*-NT, which lacks a lethal toxin [27]. The development of the concept of using microorganisms has enabled the application of genetic engineering methods. It allows equipping specific bacterial strains with genes encoding therapeutic prodrug-activating enzymes. The purpose of this review is to discuss the potential of *Clostridiaceae* bacteria in innovative anticancer therapies. This review was prepared using PubMed, Google, and ClinicalTrials.gov, with filters to focus searches on original articles, peer-reviewed studies, and clinical studies published between January 2010 and October 2025. Keywords were diverse, and the articles included in the review addressed *Clostridia* and cancer treatment.

## 2 Biological Rationale: Why *Clostridiaceae* Can Target Tumors

### 2.1 Selective Colonization of Hypoxic Areas

The best-documented and most important mechanism is the selective colonization of hypoxic areas within solid tumors. Bacteria belonging to the *Clostridiaceae* family possess a unique ability to grow selectively under strictly anaerobic conditions, enabling them to colonize deep, hypoxic areas of tumors that are inaccessible to most conventional therapies. *Clostridium* endospores, due to their selectivity, remain dormant in healthy tissues with normal oxidation, while they germinate and proliferate in the tumor environment [28]. Thus, colonization occurs only within the tumor lesion, while healthy tissues are bypassed. *Clostridium* bacteria have been shown not to colonize hypoxic non-tumor lesions [29]. Such selectivity enables the bacteria to target hard-to-reach tumor masses with hypoxic areas that standard treatment cannot eradicate, and they can serve as biological vectors to aid targeted anticancer therapy [22]. It is important to note that areas of the tumor where oxygen is completely eliminated become necrotic. These are preferential sites for the colonization, growth, and multiplication of obligate anaerobes, such as *Clostridia* species [22,27].

### 2.2 Oncolysis and Immune Activation

**Figure 1:**
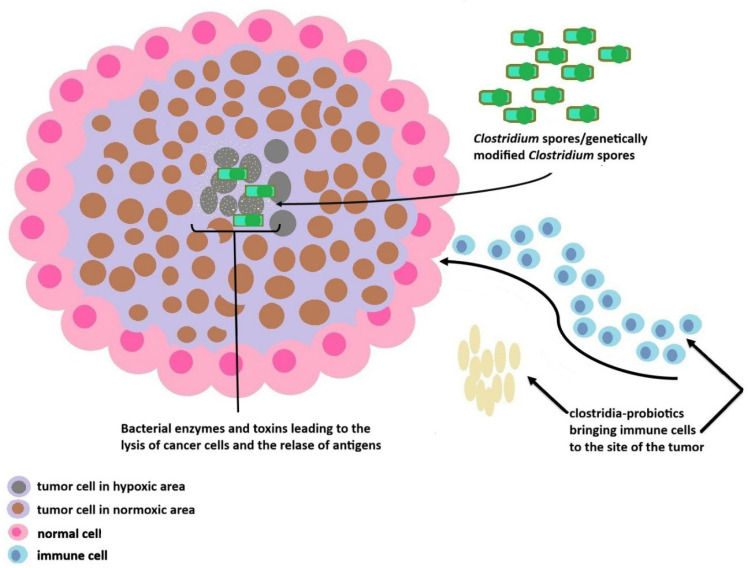
Using *Clostridium* to fight cancer. *Clostridia* can penetrate hypoxic regions within tumors. Colonizing these regions, they can lead to the lysis of tumor cells. Through this process, these cells release antigens that induce an influx of immune cells to the tumor site. This influx of these cells can also be induced by *Clostridia* probiotics.

Bacteria belonging to the *Clostridiaceae* family possess several unique biological characteristics that enable them to interact selectively with tumor tissues (Fig. 1). Their great therapeutic potential stems from a combination of several essential mechanisms, including selective colonization of hypoxic tumor zones, the ability to produce toxins and enzymes that degrade tumor tissue, and the potential to serve as biological vectors for additional therapeutic agents.

## 3 Therapeutic Modalities Using *Clostridiaceae*

### 3.1 Spore-Based Bacteriolytic Therapy

Spores of *Clostridium ghonii* exhibit great oncolytic potential and constitute an essential foundation for spore-based cancer therapy. Preclinical studies have demonstrated that *C. ghonii* spores germinate selectively in hypoxic regions of the tumor, thereby enhancing cancer cell apoptosis. This effect is attributed to *C. ghonii*’s ability to secrete collagenase IV and phospholipase C. Additionally, in animal model studies, *C. ghonii* spores reduced tumor volume compared to the control group and intensified the development of tumor necrosis areas. Moreover, *C. ghonii* synergized with radiotherapy, further potentiating tumor destruction [30]. Spore-based therapy using *C. ghonii* also activated systemic immune responses, characterized by the recruitment of T lymphocytes and increased production of INF-γ and IL-9 [31]. This multidirectional activity makes *C. ghonii* an attractive candidate for bacteriolytic cancer therapy. Positive effects of spore therapy using a non-pathogenic strain of *Clostridium ghonii* in combination with radiotherapy have also been documented. Each method used separately showed only a moderate therapeutic effect. In contrast, the use of both methods significantly increased impact, resulting in a marked reduction in tumor size, inhibition of tumor growth, and prolongation of disease-free survival. Notably, more than half of the animals experienced complete tumor regression [30].

In 41% of patients with solid tumors not amenable to standard treatment, administration of *C. novyi*-NT spores resulted in selective lytic destruction of the tumor mass. In comparison, 86% of patients achieved disease stabilization. The therapy also induced a short-term systemic increase in inflammatory cytokines and enhanced T cell responses directed against the tumor [32]. Research by Abedi Jafari et al. demonstrated that a single injection of non-toxic *Clostridium novyi* led to the complete elimination of the cancer. However, a complete cure was possible only in cases of smaller tumors, whereas larger lesions were not cured, suggesting certain limitations to this therapy [33].

### 3.2 Toxin-Based and Recombinant Toxin Approaches

The second, equally important mechanism underlying the anticancer effects of *Clostridiaceae* is the production of toxins and enzymes that can induce oncolysis of cells and the tumor’s lining. Many species of bacteria in this family can produce potent exotoxins. *C. perfringens* and *C. novyi* are known to produce the phospholipase C (alpha-toxin), which leads to degradation of the cell membrane and thus to the lysis of host cells [22].

*C. perfringens* enterotoxin (CPE), known as an agent responsible for severe food poisoning, has gained prominence as a potentially useful agent in anticancer therapy. CPE has a high affinity for the tight junction proteins claudin-3 (CLDN3) and claudin-4 (CLDN4), which act as its receptors. Their combination causes the cell membrane to disintegrate, increases its permeability by forming pores, and leads to osmotic swelling of the cell, ultimately resulting in cytolysis. This phenomenon is accompanied by a rapid influx of Ca^2+^ ions and the activation of apoptotic pathways [34,35].

In studies of colon cancer cell lines characterized by high expression of CLDN3 and CLDN4, a significant response to both the recombinant enterotoxin (recCPE) and to gene therapy using the optCPE (translation-optimized CPE-expressing vector) was observed. The ability of CPE to exert a cytotoxic effect on tumor cells involving cytolysis of the cell membrane was confirmed, further demonstrating that this process is accompanied by extensive necrosis [36].

New studies indicate that another toxin secreted by *C. perfringens*, namely epsilon toxin (Etx), exhibits cytotoxic effects on cancer cells, effectively inhibiting their growth in colorectal cancer cell lines. Its mechanism of action primarily relies on inducing oxidative stress, as evidenced by increased levels of reactive oxygen species (ROS), and it also enhances the secretion of pro-inflammatory cytokines. These effects are accompanied by cell membrane damage, decreased glutathione levels, and lipid peroxidation [37].

Anticancer effects of recombinant pore-forming alpha-toxin produced by *C. septicum* were shown in breast cancer. The entire coding sequence of α-toxin was cloned into the pET28a(+) vector and expressed as a recombinant α-toxin in *Escherichia coli*. Then, the anticancer effects of purified α-toxin against MCF-7 breast cancer cells were assessed *in vitro* and in animal models. Results showed a significant reduction in mean cancer nodule size after α-toxin treatment [38].

*Clostridioides difficile* produces toxin B (TcdB), which has inflammatory properties and can induce the secretion of cytokines and chemokines [39,40]. *C difficile* is a cause of antibiotic-related diarrhea and pseudomembranous colitis. It produces toxins, which are a virulence factor; however, studies have shown that it can be tamed and used in a cancer cell-detection approach. Especially toxin B is noteworthy because research has demonstrated that it disrupts GTPase signaling pathways, thereby influencing the regulation of cell growth and survival [41,42,43].

Recombinant toxin B significantly induced the death of breast cancer cells, inhibited cell growth, and reduced the number of S-phase cancer cells. Furthermore, it induced early and late apoptosis and decreased the level of the antiapoptotic protein Bcl-2 (B-Cell Leukaemia 2). Moreover, it inhibited tumor migration and growth, while also activating inflammation in a breast cancer model. It was also found that rcdtB significantly reduced the levels of erbB-2 (erbB-2 receptor tyrosine kinase 2) and Cox-2 (cyclooxygenase-2) in tumor tissues [38,44].

An interesting approach appears to be the use of botulinum toxin (BoNT/A1) as an adjunct to anti-PD-1 antibodies. Compared with single-agent treatment, combined anti-PD-1 and BoNT/A1 therapy significantly reduced tumor growth in mice bearing B16-F10 (melanoma) and MC38 (CRC) tumors. In a syngeneic mouse model of melanoma, combined treatment with anti-PD-1 and BoNT/A1 reduced the percentage of myeloid-derived suppressor cells (MDSCs), abolished the increase in the rate of Treg cells, and induced a greater number of CD4^+^ and CD8^+^ T lymphocytes infiltrating the tumor microenvironment compared with treatment with anti-PD-1 antibody alone [45]. The examples of *Clostridium* strains used in anticancer approaches are summarized in Table 1.

**Table 1:** Examples of *Clostridiaceae* family members in anticancer approaches.

*Clostridium*	Anticancer Approach	References
*C. butyricum*	Possible use of probiotic strains in support of anticancer therapy using immune checkpoint inhibitors	[46,47,48,49]
*C. difficile* *C. perfringens* *C. botulinum*	Toxins and their recombinant versions, which exhibit antitumor properties, are regulated through pathways related to survival and apoptosis	[22,34,35,36,37,45]
*Clostridium sporogenes* *C. novyi* *C. ghonii*	Spore therapy; possibility of using spores delivered to the tumor to eliminate tumor cells; possibility of spore modification and targeted action of *C. sporogenes* to initiate changes in the tumor microenvironment	[30,31,50,51,52]
*C. acetobutylicum*	Vector for prodrugs	[22,50,53]

### 3.3 Engineered Clostridia As Delivery Platforms

#### 3.3.1 Genetic Engineering Approaches

Initially, the concept of using *Clostridium* bacteria was to administer bacterial spores without genetic engineering. After introducing them into the body via the intravenous route, the spores were to remain inactive in healthy tissues. However, in hypoxic areas of the tumor, they would germinate, exert a local cytotoxic effect, and induce oncolysis. The first strain tested in this manner was *C. novyi*-NT, which lacked virulence factors. Thanks to its ability to move actively, this strain colonizes tissues very effectively, surpassing the colonization capabilities of other *Clostridium* strains. This distinctive ability to spread effectively into necrotic areas of the tumor is of significant importance for therapy. It has allowed *C. novyi*-NT to be selected as the most promising candidate for further research [52]. In several preclinical studies, this strain has shown antitumor activity. In a rat glioma model, intratumoral administration of *C. novyi*-NT spores resulted in selective tumor destruction without compromising the integrity of healthy brain tissue. Notably, the treatment used significantly prolonged the survival of the tested animals compared to the control group.

Studies involving dogs with naturally occurring solid tumors have shown that the applied therapy results in reproducible, strong therapeutic responses. In some cases, a significant reduction in tumor size was observed, confirming the therapeutic potential of *C. novyi*-NT. These promising results enabled the implementation of a preclinical study, which progressed to the clinical phase (NCT01924689, Table 2) [54].

*Clostridium acetobutylicum* DSM 792, which has been genetically engineered to secrete interleukin-2 (IL-2), enhances the immune response and T-cell-mediated antitumor activity. Such a strategy enabled the delivery of greater amounts of IL-2 to activate the host immune response and inhibit tumor growth, while avoiding the toxicity associated with excess systemic IL-2 [50].

The ability to genetically modify bacteria enables them to serve as carriers of anti-cancerous substances, which can be precisely delivered to tumor tissue. Modification methods include deleting genes (e.g., removing virulence genes), changing single nucleotides, overexpressing genes, or integrating novel DNA into genomes [55]. The last of these seems to be particularly interesting, especially in terms of using the CRISPR-Cas9 system.

Genetic constructs encoding toxins or enzymes are incorporated into bacterial cells lacking virulence factors. The bacteria can easily enter the tumor environment, exhibit high penetration capacity, and subvert the immune system. Once inside, they produce proteins based on engineered constructs (e.g., using CRISPR-Cas9) [56]. These may be enzymes that activate prodrugs, toxins, or immunomodulatory factors. This means the drugs are activated directly at the tumor site, increasing the treatment’s effectiveness and area-targetability [27,56]. Kubiak et al. undertook studies on the modification of *C. sporogenes*, aiming to obtain a recombinant strain that could be used in cancer therapeutics. The target genetic inserts were FMN reductase—NfrA, murine interleukin 2 (mIL-2), and murine granulocyte-macrophage colony-stimulating factor (mGM-CSF). They used two plasmids: a Cas9 expression plasmid (pTetR-PIPL12-Cas9) and a second, 6.6 kb editing plasmid (p8222F-gX-HC), containing the gRNA and repair cassette. This plasmid system increased cloning and conjugation efficiency. The introduced genetic constructs enabled the bacteria to produce cytokines. *In vitro* studies indicate the potential of this therapeutic approach. Researchers suggest that the development of precision medicine may facilitate the implementation of genetically modified *Clostridia* as highly effective cancer therapeutics [56]. Another example of CRISPR/Cas9 modification was the incorporation of the RGD tripeptide into *C. novi*-NT. The RGD (Arginine-Glycine-Aspartic Acid) protein sequence is found in many extracellular matrix proteins, enabling cell-to-cell recognition and highly efficient cell recognition and adhesion. Expression of the RGD peptide on the outer spore coat of *C*. *novyi*-NT increased the capacity for tumor localization of *C*. *novyi* upon intravenous administration based on the natural binding of RGD with the α_v_β_3_ integrin overexpressed on the epithelial tissue surrounding a tumor [57]. It leads to immune stimulation when administered intravenously in pancreatic cancer [57].

**Table 2:** Clinical trials are investigating the use of *Clostridium* bacteria in anticancer treatment.

Clinical Trial Registration Number/Phase	Cancer or Cancer-Related Disease	*Clostridia* Used in Intervention/Treatment	Purpose
NCT06855355/Not Applicable	Colorectal polyps	Dietary Supplement: *Clostridium butyricum* MIYAIRI 588	To reduce the risk of colorectal adenomatous polyp recurrence in adult patients with a history of colorectal polyps.
NCT01924689/I	Solid tumors	Intratumoral Injection of *Clostridium novyi*-NT Spores	To examine the safety of intratumoral administration of *Clostridium novyi*-NT spores in patients with treatment-refractory solid tumor malignancies.
NCT03435952/I	Solid tumors	Intratumoral Injection of *Clostridium novyi*-NT Spores	To find the highest tolerable dose of one of these bacterial therapies (*Clostridium novyi*-NT spores) that can be given in combination with pembrolizumab to patients with advanced solid tumors.
NCT06696742/II	Urothelial carcinoma	*Clostridium butyricum* tablets	To explore whether the combination of *Clostridium butyricum* can improve the current situation of poor anti-PD1 treatment effect in patients with bladder cancer.
NCT03829111/I	Renal cell carcinoma	*Clostridium butyricum* CBM 588 Probiotic Strain given PO	To determine the effect of *Clostridium butyricum* CBM 588 probiotic strain (in combination with nivolumab and ipilimumab) on the gut microbiome in patients with metastatic renal cell carcinoma (mRCC).

In addition to the CRISPR-Cas9 system, researchers also utilize other CRISPR-associated protein enzymes. Examples include studies using Cas12a, specifically Ascas12a from *Acidaminococcus* and Fncas12a from *Francisella novicida*. Zhang et al. demonstrated highly efficient and rapid genome modification using Cas12a-mediated systems, as evidenced by the effective introduction of the CRISPR-Cas12a system into two *Clostridium* sensu stricto species, *C. butyricum*, and *C. sporogenes*, utilizing tetracycline-inducible systems [58]. Another study on Clostridial concerns, using CRISPR-FnCas12a with a multiplex gene-knockout approach. An attempt was made to simultaneously knock out several genes using Cas12a in *Clostridium beijerinckii* [59]. It turned out that this approach could generate modified bacteria, but the effectiveness of gene switching varied from 25 to 100% [59]. On the one hand, this indicates the great potential of genetic engineering to produce bacteria with desirable properties. On the other hand, the uneven efficiency of gene deactivation indicates the need for further research to improve CRISPR/Cas techniques.

**Figure 2:**
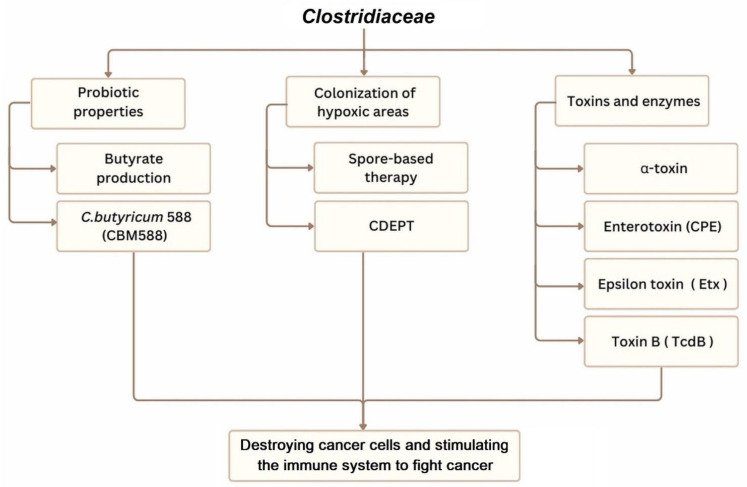
Directions for *Clostridiaceae* action in the anticancer approach. CDEPT—Directed Enzyme Prodrug Therapy; CPE—*C. perfringens* enterotoxin; Etx—*C. perfringens*, epsilon toxin, TcdB—*Clostridioides difficile* toxin B Abb.

Fig. 2 presents the directions of action of *Clostridium* bacteria in the context of the anticancer approach, highlighting their influence on the immune response and the immune system’s anticancer activity.

#### 3.3.2 Clostridia-Directed Enzyme Prodrug Therapy (CDEPT)

To enhance the effectiveness of the therapy, strategies have been developed to engineer genetically *Clostridium* bacteria. A promising approach appears to be *Clostridium*-Directed Enzyme Prodrug Therapy (CDEPT), in which modified *Clostridium* spores populate the tumor environment and then produce enzymes that activate prodrugs and convert them into cytotoxic forms [53].

These modifications fall under the DEPT (Directed Enzyme Prodrug Therapy) methods, which deliver prodrug-activating enzymes directly to the tumor site. CDEPT, is an innovative and experimental approach that uses genetically modified *Clostridium* anaerobic bacteria. Injected into the body, the spores germinate only in necrotic areas of the tumor, where they are activated and express an exogenous enzyme that converts a harmless prodrug into its active form, which has cytostatic activity [60]. Restricting the enzyme’s activity exclusively to the tumor area reduces its potential toxic effects on healthy tissues. In CDEPT therapy, there are two primary enzyme-drug compounds. One is cytosine deaminase (CD), and the other is 5-fluorocytosine (5-FC). CD catalyzes the conversion of 5-FC to 5-fluorouracil (5-FU), which is a potent anticancer drug. In preclinical studies using a genetically modified strain of *C. sporogenes* engineered to express CD, the effectiveness of such targeted expression of the enzyme on tumor areas was confirmed. After administering such modified spores to test animals with solid tumors, bacterial colonization in the tumor environment and, most importantly, significant CD production were observed. The additional application of the prodrug 5-FC, which is converted to the cytotoxic 5-FU in the presence of CD, resulted in substantial inhibition of tumor growth [50].

Activation of the prodrug can also be catalyzed by NADPH (Nicotinamide Adenine Dinucleotide Phosphatase)-dependent quinone reductases, classified into two prominent protein families in the Pfam database: Pfam PF00881 Nitroreductase and Pfam PF02525. The distinguished representatives of these families, most often studied in the context of CDEPT therapy, are NfnB from *Escherichia coli* and YwrO from *Bacillus amyloliquefaciens*, which can activate the anticancer prodrug tretazicar (CB1954) [60,61].

Although the CB1954 prodrug showed antitumor activity in monotherapy in preclinical studies, its use was associated with significant side effects. In contrast, the introduction of *E. coli* nitroreductase into *C. sporogenes* enabled the safe use of CB1954 and the newer prodrug PR-104. Their combination resulted in marked tumor growth inhibition and significantly greater efficacy, with no observed toxicity [62]. A novel nitroreductase (NmeNTR) derived from *Neisseria meningitidis* was also isolated and introduced into *C. sporogenes* to enhance the therapeutic efficacy. It enabled the creation of a new, safe strain that more efficiently activates the CB1954 prodrug in a clinical setting [60]. A significant antitumor effect was also obtained when nitroreductase expressed by *C. sporogenes* was combined with the prodrug PR-104 [50].

## 4 *Clostridium* in Combination with Conventional Treatments

Due to the limitations of the independent action of bacteria, such as *C. novyi*-NT, among others, in well-oxygenated areas of the tumor marginal zone, combination therapies appear to be a promising approach. Improved efficacy and increased treatment response can be achieved by integrating the oncolytic and immune potential of *Clostridium* with standard therapies such as chemotherapy and radiotherapy. The combination of conventional treatments, such as chemotherapy or radiotherapy, with a bacteriolytic therapeutic strategy is referred to as combined bacteriolytic therapy (COBALT) [63]. For example, a study in a mouse model of cervical cancer showed that treatment with *C. novyi*-NT significantly increased the efficacy of chemotherapy. The combination of intraperitoneally administered cisplatin and intratumorally administered *C. novyi*-NT spores provided significant benefits compared with chemotherapy alone. This combination reduced markers of hypoxia and angiogenesis, specifically HIF-1α and VEGF (Vascular Endothelial Growth Factor) proteins, further decreasing the mitotic index of tumor cells [64].

Other preclinical studies have demonstrated that *C. novyi*-NT, when combined with classical cytostatics such as mitomycin-C or dolastin-10, results in extensive tumor necrosis and significantly improved antitumor effects compared with chemotherapy alone. In contrast, the combination of bacteria with microtubule stabilizers or liposomal doxorubicin resulted in considerable tumor regression, in some cases even leading to complete remission [63].

Similar effects were obtained after combining targeted therapy with *C. butyricum*. In a xenograft model of CRC (colorectal cancer) in mice, a marked delay in tumor growth was observed after combination therapy with apatinib (selective vascular endothelial growth factor receptor inhibitor) and *C. butyricum*. Importantly, in mice receiving this combination, the level of C31, an endothelial marker of angiogenesis, was significantly lower than in mice receiving monotherapy [65]. Proposed by Zhang et al., a therapeutic strategy combining irreversible electroporation (IRE) with *C. novyi*-NT may represent a practical approach for treating colorectal cancer liver metastases (CRLM). IRE uses electrical pulses to damage cancer cell membranes, leading to their death, and to disrupt tumor microcirculation, inducing transient hypoxia within the tumor. This modified tumor environment enables more effective dissemination and germination of *Clostridium* spores, enhancing their cytolytic activity. Additionally, both strategies activate the immune response [66].

## 5 Probiotic *Clostridia* and Microbiome-Mediated Support

### 5.1 Anticancer Mechanisms of Probiotic Clostridia

Particular probiotic *Clostridium* species, such as *Clostridium butyricum*, play an essential role in modulating immune responses and influencing the efficacy of anticancer therapies. *C. butyricum* is the leading producer of butyrate, a short-chain fatty acid (SCFA). Animal studies have demonstrated that a high-fat diet significantly increases the risk of intestinal tumor development, whereas *C. butyricum* inhibits tumor growth by reducing cellular proliferation, promoting apoptosis, and activating SCFA-dependent receptors GPR43 and GPR109A (G-protein-coupled receptors) [46].

Studies have also shown that *C. butyricum* modulates pathways associated with epithelial-mesenchymal transition (EMT). Downregulation of METTL3 (methyltransferase 3), a protein strongly associated with tumor progression, increased E-cadherin levels while simultaneously reducing the concentrations of mesenchymal markers such as vimentin and N-cadherin. These changes reduced cancer cell migration and invasiveness by suppressing EMT, a key process in metastasis formation [47].

The probiotic strain of *C. butyricum* has demonstrated anticancer activity not only in solid tumors but also in hematologic malignancy models. The study by Konishi et al. showed that components derived from the *C. butyricum* culture supernatant significantly reduced the viability of myeloma cell lines, and the cytotoxic effects were attributed to the butyrate produced by this strain. In *in vivo* studies, butyrate exhibited a dose-dependent effect and greater toxicity toward cancer cells. The anticancer activity of *C.butyricum*-derived butyrate was associated with its ability to induce apoptosis, as evidenced by G1 cell-cycle arrest, an increased sub-G1 population, elevated cleaved PARP (poly(ADP-ribose) polymerase) levels, and a higher proportion of Annexin V-positive cells [48].

An effective probiotic nanosystem has been developed, consisting of liposomes containing a chemotherapeutic agent conjugated to a *C. butyricum* strain via a peptide sensitive to metalloproteinase 2 (MMP-2). In the first stage, the nanosystem containing the TGF-β receptor inhibitor (Vactosertib) targeted pancreatic stellate cells (PSCs) and inhibited their activity. It led to loosening of the ECM (Extracellular matrix) and improved gemcitabine (GEM) penetration in the tumor using the second nanosystem. The use of this probiotic nanosystem significantly enhanced the penetration of chemotherapeutic agents into the tumor, while CB regulated the growth of γ-proteobacteria responsible for GEM degradation. Although this combination of chemotherapy and bacterial therapy still requires extensive safety testing, it appears to be a promising and innovative approach to the treatment of pancreatic cancer [67].

### 5.2 Probiotics As Immunotherapy Adjuvants

The beneficial effects of *C. butyricum* have also been linked to its ability to remodel the gut microbiota. Studies have reported increases in the number of health-promoting bacterial strains and reductions in pathogenic strains [46]. Through the interaction between the bacterial surface protein SecD and the GPR78 receptor expressed on colorectal cancer cells, *C. butyricum* can locally modulate the tumor microenvironment. This treatment decreased IL-6 production and increased the cytotoxic activity of CD8^+^ lymphocytes, resulting in an intensified antitumor response. Significantly, in animal model studies, this probiotic strain increased the efficacy of anti-PD-1 therapy. Notably, *C. butyricum* was able to overcome resistance to anti-PD-1 (Programmed Death 1) treatment in tumor types that generally do not respond to immune checkpoint blockade [49].

Tajima et al. demonstrated that the combination of the probiotic CBM588 and levofloxacin (LVFX) enhanced the efficacy of anti-PD-1 therapy in a colorectal cancer model, associated with increased CD8^+^ lymphocyte activity. The synergistic action of LVFX, CBM588, and anti-PD-1 led to tumor growth inhibition and a higher number of complete responses (CR), a beneficial effect that results exclusively from the specific combination of these agents. Additionally, the findings suggest that selecting appropriate probiotics, such as CBM588, may prevent the negative impact of antibiotics on the effectiveness of immune checkpoint inhibitor therapy [68].

At the molecular level, CB CM (*C. butyricum* conditioned medium) enhances ubiquitination and degradation of the MYC (MYC Proto-Oncogene, BHLH Transcription Factor) oncoprotein. It results in the downregulation of thymidylate synthase (TYMS) expression, which is associated with resistance to 5-FU treatment, and increases the sensitivity of CRC cells to chemotherapy. Moreover, CB may enhance the efficacy of anti-PD-1 immunotherapy. Studies of both live bacillus and CB CM alone showed slower tumor growth and increased infiltration of CD4^+^ and CD8^+^ lymphocytes, accompanied by increased granzyme-B expression, compared with anti-PD-1 monotherapy. These results confirm CB’s ability to enhance anti-PD-1 immunotherapy by modulating the immune response [69].

Studies in mice have shown that CBM588 supplementation improves gut homeostasis and effectively overcomes resistance to PD-1 blockade in non-small cell lung cancer. Furthermore, at the level of the tumor-draining lymph node, this treatment combination significantly reduced the frequency of a microbiota-modulated subset of regulatory T cells that express the orphan retinoic acid receptor gamma T (Rorγt^+^ Treg). A reduced intratumoral accumulation of immunosuppressive RORγt^+^ Treg influenced the enhanced effectiveness of PD-1 blockade [70].

## 6 Clinical Evidence and Ongoing Clinical Trials

Research on combination therapy has entered phase I clinical trials (NCT03435952), in which researchers at the M.D. Anderson Cancer Center evaluated the safety and efficacy of combining *C. novyi*-NT spores with pembrolizumab in patients with advanced solid tumors.

The therapy demonstrated a favorable safety profile with only minor side effects. Notably, a 25% objective response rate was observed, including three partial responses and one complete response, and disease stabilization was confirmed in 69% of treated patients [71].

An example of the use of *C. butyricum* in clinical trials is NCT06855355, which evaluates the preventive efficacy of this strain and includes a group of patients after polyp removal. The assessment of polyp recurrence following supplementation with *C. butyricum* MIYAIRI 588 (CBM588) in the study participants may be of significant importance for colorectal cancer prevention, indicating a high potential to reduce the risk of tumor recurrence. In contrast, the NCT06696742 trial aims to assess the impact of *C. butyricum* administration on the efficacy of PD-1 inhibitors in patients with urothelial cancer.

Promising results were obtained in the NCT03829111 study, which included patients with metastatic renal cell carcinoma. In this trial, CBM588 was introduced in patients receiving the combination of nivolumab and ipilimumab. This group demonstrated prolonged progression-free survival and an increased response rate. Immune system activation analysis showed elevated levels of IL-1β, IL-10, IL-12, IL-2, G-CSF (Granulocyte-Colony Stimulating Factor), and TNF-α. These findings suggest that adding CBM588 to therapy may enhance the immune response and support the use of immune checkpoint inhibitors [72]. It should be mentioned that they do not kill cells directly, but indicate the site of action of the immune system.

## 7 Limitations and Future Perspective

There is a gap between the bench and the bedside in implementing *Clostridia* treatment in everyday clinical practice. Despite numerous advantages and promising results from experimental studies (*in vitro* and *in vivo* in animals), therapies based on *Clostridiaceae* have not been introduced into routine clinical practice due to multiple barriers.

One significant challenge associated with these approaches is safety. Several *Clostridiaceae* species are pathogenic, and even when attenuated strains are used, there remains a risk of adverse events such as sepsis, gas gangrene, infections, or skin rashes [32].

Still are conducting research with attenuated strains lacking virulence factors. An example of such a strain is *Clostridium novyi*-NT, which has been stripped of the alpha-toxin gene yet retains the ability to colonize tumors and kill tumor cells through the production of other enzymes, such as phospholipases and lipolytic enzymes [27]. Even if they are genetically modified, they may be unstable in standing constructs and acquire virulence traits. It is crucial to eliminate the possibility that modified therapeutic *Clostridiaceae* strains acquire virulence or antibiotic resistance. Therapeutic strains must not be capable of horizontal transfer of genetic material, a characteristic of *Clostridiaceae*. This is one of the safety aspects of the potential use of these bacteria in the treatment of oncology patients. From this perspective, genetic engineering will undoubtedly be a very important branch of the process of developing safe clostridia-based therapeutics. Safety of use is a key aspect of introducing *Clostridial* therapy and is also related to the current gap between research and practical application.

The challenge is to control bacterial proliferation after tumor colonization, prevent systemic toxicity, and maximize therapeutic effectiveness. Strategies to manage this proliferation include antibiotic intervention. Metronidazole can be used to kill vegetative Clostridial cells. Further, Genetic engineering methods can introduce not only therapeutic constructs but also regulatory elements that will determine the production of therapeutic agents only at precisely defined sites, i.e., in areas of hypoxia or tumor necrosis [73]. Therefore, attempts are being made to incorporate an HRE (hypoxia-responsive element), a specific cis-acting DNA sequence located within the promoter or enhancer regions of genes that respond to hypoxia.

Studies also indicate that tumor size may significantly limit the effectiveness of the *Clostridial* therapeutic strategy. The therapy may not be effective in small tumors or metastatic lesions that lack hypoxic or necrotic regions. Furthermore, *Clostridiaceae* therapy may be ineffective in marginal, oxygenated, and potentially invasive areas. Research has demonstrated that in small breast tumors, treatment with *C. novyi*-NT led to substantial tumor mass reduction and complete regression in experimental animals. In contrast, larger tumors did not similarly respond to *C. novyi*-NT therapy, suggesting that this monotherapy is insufficient and likely requires combination with additional treatment modalities [33].

One significant clinical limitation of bacteriolytic therapy is the interaction between the administered bacteria and the host immune system. Preclinical studies have shown that the administration of *C. novyi*-NT spores triggers rapid recruitment of white blood cells, predominantly neutrophils, as a natural defense response to germinating spores, followed by their subsequent migration into tumor tissue. The accumulating immune cells form a barrier that prevents the bacteria from spreading throughout the tumor, thereby weakening the overall bacteriolytic effect. Therefore, therapeutic strategies employing lytic bacteria should consider modulating the host immune response to enhance the therapeutic potential of this approach [74].

## 8 Conclusion

The use of bacteria from the *Clostridiaceae* family opens new therapeutic possibilities. *Clostridium* strains are already being used in clinical trials. The positive results of these studies will allow the introduction of alternative therapies based on the natural action of anaerobic bacterial metabolites, in addition to conventional, debilitating ones. It is crucial, especially considering the projected future increase in the number of cancer patients. Microbiological therapies that regulate intestinal function, which in turn determine the proper state of the immune system, and the use of bacterial metabolites to reduce tumor growth appear to be a challenge for researchers and an opportunity for cancer patients in the future.

## Data Availability

Not applicable.
